# Solutions of a Two-Particle Interacting Quantum Walk

**DOI:** 10.3390/e20060435

**Published:** 2018-06-05

**Authors:** Alessandro Bisio, Giacomo Mauro D’Ariano, Nicola Mosco, Paolo Perinotti, Alessandro Tosini

**Affiliations:** Dipartimento di Fisica dell’Università di Pavia, Istituto Nazionale di Fisica Nucleare, Pavia 27100, Italy

**Keywords:** quantum walks, Hubbard model, Thirring model

## Abstract

We study the solutions of an interacting Fermionic cellular automaton which is the analogue of the Thirring model with both space and time discrete. We present a derivation of the two-particle solutions of the automaton recently in the literature, which exploits the symmetries of the evolution operator. In the two-particle sector, the evolution operator is given by the sequence of two steps, the first one corresponding to a unitary interaction activated by two-particle excitation at the same site, and the second one to two independent one-dimensional Dirac quantum walks. The interaction step can be regarded as the discrete-time version of the interacting term of some Hamiltonian integrable system, such as the Hubbard or the Thirring model. The present automaton exhibits scattering solutions with nontrivial momentum transfer, jumping between different regions of the Brillouin zone that can be interpreted as Fermion-doubled particles, in stark contrast with the customary momentum-exchange of the one-dimensional Hamiltonian systems. A further difference compared to the Hamiltonian model is that there exist bound states for every value of the total momentum and of the coupling constant. Even in the special case of vanishing coupling, the walk manifests bound states, for finitely many isolated values of the total momentum. As a complement to the analytical derivations we show numerical simulations of the interacting evolution.

## 1. Introduction

Quantum walks (QWs) describe the evolution of one-particle quantum states on a lattice, or, more generally, on a graph. The quantum walk evolution is linear in the quantum state and the quantum aspect of the evolution occurs in the interference between the different paths available to the walker. There are two kinds of quantum walks: continuous time QWs, where the evolution operator of the system given in terms of an Hamiltonian can be applied at any time (see Farhi et al. [1]), and discrete-time QWs, where the evolution operator is applied in discrete unitary time-steps. The discrete-time model, which appeared already in the Feynman discretization of the Dirac equation [2], was later rediscovered in quantum information [3,4,5,6,7], and proved to be a versatile platform for various scopes. For example, QWs have been used for empowering quantum algorithms, such as database search [8,9], or graph isomorphism [10,11]. Moreover, quantum walks have been studied as a simulation tool for relativistic quantum fields [12,13,14,15,16,17,18,19,20,21,22,23,24,25,26,27,28], and they have been used as discrete models of spacetime [29,30,31,32].

QWs are among the most promising quantum simulators with possible realizations in a variety of physical systems, such as nuclear magnetic resonance [33,34], trapped ions [35], integrated photonics, and bulk optics [36,37,38,39].

New research perspectives are unfolding in the scenario of multi-particle interacting quantum walks where two or more walking particles are coupled via nonlinear (in the field) unitary operators. The properties of these systems are still largely unexplored. Both continuous-time [40] and discrete-time [41] quantum walks on sparse unweighted graphs are equivalent in power to the quantum circuit model. However, it is highly non-trivial to design a suitable architecture for universal quantum computation based on quantum walks. Within this perspective, a possible route has been suggested in [42] based on interacting multi-particle quantum walks with indistinguishable particles (Bosons or Fermions), proving that “almost any interaction” is universal. Among the universal interacting many-body systems are the models with coupling term of the form $\chi \delta_{x_{1},x_{2}}\hat{n}\left(x_{1}\right)\hat{n}\left(x_{2}\right)$, with $\hat{n}\left(x\right)$ the number operator at site *x*. The latter two-body interaction lies at the basis of notable integrable quantum systems in one space dimension such as the Hubbard and the Thirring Hamiltonian models.

The first attempts at the analysis of interacting quantum walks were carried out in [43,44]. More recently, in [45], the authors proposed a discrete-time analogue of the Thirring model, which is indeed a Fermionic quantum cellular automaton, whose dynamics in the two-particle sector reduces to an interacting two-particle quantum walk. As for its Hamiltonian counterpart, the discrete-time interacting walk has been solved analytically in the case of two Fermions. Analogously to any Hamiltonian integrable system, also in the discrete-time case the solution is based on the Bethe Ansatz technique. However, discreteness of the evolution prevents the application of the usual Ansatz, and a new Ansatz has been introduced successfully [45].

In this paper, we present an original simplified derivation of the solution of [45], which exploits the symmetries of the interacting walk. We present the diagonalization of the evolution operator and the characterization of its spectrum. We explicitly write the two particle states corresponding to the scattering solutions of the system, having eigenvalues in the continuous spectrum of the evolution operator. We then show how the present model predicts the formation of bound states, which are eigenstates of the interacting walk corresponding to the discrete spectrum. We provide also in this case the analytic expression of such molecular states.

We comment on the phenomenological differences between the Hamiltonian model and the discrete-time one. First, we see that the set of possible scattering solutions is larger in the discrete-time case: for a fixed value total momentum, a non trivial transfer of relative momentum can occur besides the simple exchange of momentum between the two particles, differently from the Hamiltonian case. In addition, the family of bound states appearing in the discrete-time scenario is larger than the corresponding Hamiltonian one. Indeed, for any fixed value of the coupling constant, a bound state exists with any possible value of the total momentum, while, for Hamiltonian systems, bound states cannot have arbitrary total momentum.

Finally, we show that, in the set of solutions for the interacting walk, there are perfectly localized states (namely, states that lie on a finite number of lattice sites). Moreover, differently from the Hamiltonian systems, bound states exist also for null coupling constants; however, this is true only for finitely many isolated values of the total momentum. In addition to the exact analytical solution of the dynamics, we show the simulation of some significant initial states.

## 2. The Dirac Quantum Walk

In this section, we review the Dirac quantum cellular automaton on the line describing the free evolution of a two-component Fermionic field. The single particle Hilbert space is given by $H:=C^{2}⊗\ell^{2}\left(Z\right)$ for which we employ the factorized basis |a〉|x〉, with a∈{↑,↓} and x∈Z. The Dirac automaton describes an arbitrary number of Fermions whose evolution is linear in the field:

(1) $$\begin{array}{c} \psi \left(x,t+1\right)=W\psi \left(x,t\right),\;\psi \left(x,t\right)=\left(\begin{array}{c} \psi_{↑}\left(x,t\right)\\ \psi_{↓}\left(x,t\right) \end{array}\right), \end{array}$$

(2) $$\begin{array}{c} {}[\psi_{a}\left(x\right),\psi_{b}^{†}\left(x^{\prime }\right){]}_{+}=\delta_{a,b}\delta_{x,x^{\prime }},\;{\left[\psi_{a}\left(x\right),\psi_{b}\left(x^{\prime }\right)\right]}_{+}=0, \end{array}$$

where W is a unitary operator. In the single particle sector, the automaton can be regarded as a quantum walk on the single-particle Hilbert space H whose evolution unitary operator *W* is given by

(3) $$W=\left(\begin{array}{cc} \nu T_{x} & -i\mu \\ -i\mu & \nu T_{x}^{†} \end{array}\right),\;\nu ,\mu >0,\;\nu^{2}+\mu^{2}=1,$$

where $T_{x}$ denotes the translation operator on $\ell^{2}\left(Z\right)$, defined by $T_{x}|x\rangle =|x+1\rangle$.

Since the walk *W* is translation invariant (it commutes with the translation operator), it can be diagonalized in momentum space. In the momentum representation, defining $|p\rangle :={\left(2\pi \right)}^{-1/2}\sum_{x\in Z}e^{-ipx}|x\rangle$, with p∈B:=(−π,π], the walk operator can be written as

(4) $$\begin{array}{c} W=\int_{B}\;\;dp\;W\left(p\right)⊗|p\rangle \;\langle p|,\;W\left(p\right)=\left(\begin{array}{cc} \nu e^{ip} & -i\mu \\ -i\mu & \nu e^{-ip} \end{array}\right), \end{array}$$

where ${\left|\nu \right|}^{2}+{\left|\mu \right|}^{2}=1$. The spectrum of the walk is given by $\left\{e^{-i\omega (p)},\;e^{i\omega (p)}\right\}$, where the dispersion relation ω(p) is given by

(5) ω(p):=Arccos(νcosp),

where Arccos denotes the principal value of the arccosine function. The single-particle eigenstates, solving the eigenvalue problem

(6) $$\begin{array}{c} W\left(p\right)v_{p}^{s}=e^{-is\omega (p)}v_{p}^{s}\;,\;s=\pm , \end{array}$$

can be conveniently written as

(7) $$\begin{array}{c} v_{p}^{s}=\frac{1}{|N_{s}|}\left(\begin{array}{c} -i\mu \\ g_{s}\left(p\right) \end{array}\right), \end{array}$$

with $g_{s}\left(p\right):=-i\left(s\sin\omega \left(p\right)+\nu \sin p\right)$, $|N_{s}{|}^{2}:=\mu^{2}+{\left|g_{s}\right|}^{2}$.

## 3. The Thirring Quantum Walk

In this section, we present a Fermionic cellular automaton in one spatial dimension with an on-site interaction, namely two particles interact only when they lie at the same lattice site. The linear part corresponds to the Dirac QW [17] and the interaction term is the most general number-preserving coupling in one dimension [46]. The same kind of interaction characterizes also the most studied integrable quantum systems, such as the Thirring [47] and the Hubbard [48] models.

The linear part of the automaton is given by the Dirac automaton, describing the free evolution of the particles. In order to introduce an interaction, we modify the evolution operator adding an extra unitary step of the form:

(8) $$V_{\mathrm{int}}:=e^{i\chi n_{↑}\left(x\right)n_{↓}\left(x\right)},$$

where $n_{a}\left(x\right)$, a∈{↑,↓}, represents the particle number at site *x*, namely $n_{a}\left(x\right)=\psi_{a}^{†}\left(x\right)\psi_{a}\left(x\right)$, and χ is a real coupling constant. Since the interaction term preserves the total number operator, we can study the automaton for a fixed number of particles. For *N* interacting particles, we can describe the evolution in terms of an interacting quantum walk over $H_{N}=H^{⊗N}$ with the free evolution given by $W_{N}:=W^{⊗N}$.

In this work, we focus on the two-particle sector whose solutions has been derived in [45]. As we will see, the Thirring walk features molecule states besides scattering solutions. This features is shared also by the Hadamard walk with the same on-site interaction [44].

$W_{N}:=W^{⊗N}$, acting on the Hilbert space $H_{N}=H^{⊗N}$ and describing the free evolution of the particles. In order to introduce an interaction, we modify the update rule of the walk with an extra step $V_{\mathrm{int}}$: $U_{N}:=W_{N}V_{\mathrm{int}}$. In the present case, the term $V_{\mathrm{int}}$ has the form

(9) $$V_{\mathrm{int}}=V_{N}\left(\chi \right):=e^{i\chi n_{↑}\left(x\right)n_{↓}\left(x\right)}.$$

Since we focus on the solutions involving the interaction of two particles, it is convenient to write the walk in the centre of mass basis $|a_{1},a_{2}\rangle |y\rangle |w\rangle$, with $a_{1},\;a_{2}\in \left\{↑,↓\right\}$, $y=x_{1}-x_{2}$ and $w=x_{1}+x_{2}$. Therefore, on this basis, the generic Fermionic state is $|\psi \rangle =\sum_{a_{1},a_{2},y,w}c\left(a_{1},a_{2},y,w\right)|a_{1},a_{2}\rangle |y\rangle |w\rangle$ with $c\left(a_{2},a_{1},y,w\right)=-c\left(a_{1},a_{2},-y,w\right)$. Notice that only the pairs y,w with *y* and *w*, both even or odd, correspond to physical points in the original basis $x_{1},x_{2}$.

We define the two-particle walk with both *y* and *w* in Z, so that the linear part of walk can be written as

(10) $$\begin{array}{c} W_{2}=\mu \nu \left(\begin{array}{cccc} \frac{\nu }{\mu }T_{w}^{2} & -iT_{y}⊗T_{w} & -iT_{y}^{†}⊗T_{w} & -\frac{\mu }{\nu }\\ -iT_{y}⊗T_{w} & \frac{\nu }{\mu }T_{y}^{2} & -\frac{\mu }{\nu } & -iT_{y}^{†}⊗T_{w}\\ -iT_{y}^{†}⊗T_{w} & -\frac{\mu }{\nu } & \frac{\nu }{\mu }{T_{y}^{†}}^{2} & -iT_{y}^{†}⊗T_{w}^{†}\\ -\frac{\mu }{\nu } & -iT_{y}⊗T_{w}^{†} & -iT_{y}^{†}⊗T_{w}^{†} & \frac{\nu }{\mu }{T_{w}^{†}}^{2} \end{array}\right), \end{array}$$

where $T_{y}$ represents the translation operator in the relative coordinate *y*, and $T_{w}$ the translation operator in the centre of mass coordinate *w*, whereas the interacting term reads

(11) $$V_{2}\left(\chi \right)=\left(\begin{array}{cccc} I_{y}⊗I_{w} & 0 & 0 & 0\\ 0 & e^{i\chi \delta_{y,0}}⊗I_{w} & 0 & 0\\ 0 & 0 & e^{i\chi \delta_{y,0}}⊗I_{w} & 0\\ 0 & 0 & 0 & I_{y}⊗I_{w} \end{array}\right).$$

This definition gives a walk $U_{2}=W_{2}V_{2}\left(\chi \right)$ that can be decomposed into two identical copies of the original walk. Indeed, defining as *C* the projector on the physical center of mass coordinates, one has $U_{2}=CU_{2}C+\left(I-C\right)U_{2}\left(I-C\right)$, where $CU_{2}C$ and $\left(I-C\right)U_{2}\left(I-C\right)$ are unitarily equivalent. We will then diagonalize the operator $U_{2}$, reminding readers that the physical solutions will be given by projecting the eigenvectors with *C*.

Introducing the (half) relative momentum $k=\frac{1}{2}\left(p_{1}-p_{2}\right)$ and the (half) total momentum $p=\frac{1}{2}\left(p_{1}+p_{2}\right)$, the free evolution of the two particles is written in the momentum representation as

(12) $$\begin{array}{c} W_{2}=\int \;\;dkdp\;W_{2}\left(p,k\right)⊗|k\rangle \;\langle k|⊗|p\rangle \;\langle p|, \end{array}$$

where the matrix $W_{2}\left(p,k\right)$ is given by

(13) $$\begin{array}{c} W_{2}\left(p,k\right)=W\left(p_{1}\right)⊗W\left(p_{2}\right). \end{array}$$

Furthermore, we introduce the vectors $v_{k}^{sr}:=v_{p+k}^{s}⊗v_{p-k}^{r}$, with s,r=±, such that

(14) $$\begin{array}{c} W_{2}\left(p,k\right)v_{k}^{sr}=e^{-i\omega_{sr}\left(p,k\right)}v_{k}^{sr}, \end{array}$$

where $\omega_{sr}\left(p,k\right):=s\omega \left(p+k\right)+r\omega \left(p-k\right)$ is the dispersion relation of the two-particle walk. Explicitly, the vectors $v_{k}^{sr}$ are given by

(15) $$v_{k}^{sr}=\frac{1}{|N_{s}\left(p+k\right)|\;|N_{r}\left(p-k\right)|}\left(\begin{array}{c} -\mu^{2}\\ -i\mu g_{r}\left(p-k\right)\\ -i\mu g_{s}\left(p+k\right)\\ g_{s}\left(p+k\right)g_{r}\left(p-k\right) \end{array}\right).$$

We focus in this work on Fermionic solutions satisfying the eigenvalue equation

(16) $$U_{2}\left(\chi ,p\right)|\psi \rangle =e^{-i\omega }|\psi \rangle ,\;\omega \in R,$$

with $|\psi (y)\rangle \in C^{4}$. In the centre of mass basis, the antisymmetry condition reads

(17) $$\begin{array}{c} |\psi (y)\rangle =-E|\psi (-y)\rangle , \end{array}$$

*E* being the exchange matrix

(18) $$\begin{array}{c} E=\left(\begin{array}{cccc} 1 & 0 & 0 & 0\\ 0 & 0 & 1 & 0\\ 0 & 1 & 0 & 0\\ 0 & 0 & 0 & 1 \end{array}\right). \end{array}$$

## 4. Symmetries of the Thirring Quantum Walk

The Thirring walk manifests some symmetries that allow for simplifying the derivation and the study of the solutions. First of all, as we already mentioned, one can show that the interaction V(χ) commutes with the total number operator. This means that one can study the walk dynamics separately for each fixed number of particles. We focus here on the two-particle walk $U_{2}=W_{2}V_{2}\left(\chi \right)$, where $W_{2}=W⊗W$ and $V_{2}\left(\chi \right)=e^{i\chi \delta_{y,0}\left(1-\delta_{a_{1},a_{2}}\right)}$.

Since the interacting walk $U_{2}$ commutes with the translations in the centre of mass coordinate *w*, the total momentum is a conserved quantity, so it is convenient to study the walk parameterized by the total momentum *p*. To this end, we consider the basis $|a_{1},a_{2}\rangle |y\rangle |p\rangle$, so that, for fixed values of *p*, the interacting walk of two particles can be expressed in terms of a one-dimensional QW $U_{2}\left(\chi ,p\right)=W_{2}\left(p\right)V\left(\chi \right)$ with a four-dimensional coin:

(19) $$\begin{array}{c} W_{2}\left(p\right)=\mu \nu \left(\begin{array}{cccc} \frac{\nu }{\mu }e^{i2p} & -ie^{ip}T_{y} & -ie^{ip}T_{y}^{†} & -\frac{\mu }{\nu }\\ -ie^{ip}T_{y} & \frac{\nu }{\mu }T_{y}^{2} & -\frac{\mu }{\nu } & -ie^{-ip}T_{y}\\ -ie^{ip}T_{y}^{†} & -\frac{\mu }{\nu } & \frac{\nu }{\mu }{T_{y}^{†}}^{2} & -ie^{-ip}T_{y}^{†}\\ -\frac{\mu }{\nu } & -ie^{-ip}T_{y} & -ie^{-ip}T_{y}^{†} & \frac{\nu }{\mu }e^{-i2p} \end{array}\right). \end{array}$$

Although the range of the variable *p* is the interval (−π,π], it is possible to show that one can restrict the study of the walk to the interval [0,π/2]. On the one hand, the two-particle walk transforms unitarily under a parity transformation in the momentum space. Starting from the single particle walk, W(p) transforms under a parity transformation as

(20) $$\begin{array}{c} W\left(p\right)=\sigma_{x}W\left(-p\right)\sigma_{x},\;p\in \left(-\pi ,\pi \right], \end{array}$$

so that, for the two-particle walk, we have the relation

(21) $$W_{2}\left(-p,y\right)=\sigma_{x}⊗\sigma_{x}\;EW_{2}\left(p,y\right)E\;\sigma_{x}⊗\sigma_{x}.$$

On the other hand, a translation of π of the total momentum *p* entails that

(22) $$\begin{array}{c} W_{2}\left(p+\pi ,y\right)=\sigma_{z}⊗\sigma_{z}\;W_{2}\left(p,y\right)\;\sigma_{z}⊗\sigma_{z}, \end{array}$$

while the interaction term remains unaffected in both cases.

The Thirring walk features also another symmetry that can be exploited to simplify the derivation of the solutions. It is easy to check that the walk operator $U_{2}\left(p,\chi \right)=W_{2}\left(p\right)V\left(\chi \right)$ commutes with the projector defined by

(23) $$P:=\left(\begin{array}{cccc} P_{o} & 0 & 0 & 0\\ 0 & P_{e} & 0 & 0\\ 0 & 0 & P_{e} & 0\\ 0 & 0 & 0 & P_{o} \end{array}\right),$$

where $P_{e}$ and $P_{o}$ are the projectors on the even and the odd subspaces, respectively:

(24) $$\begin{array}{c} P_{e}=\sum_{z\in Z}|2z\rangle \;\langle 2z|,\;P_{o}=\sum_{z\in Z}|2z+1\rangle \;\langle 2z+1|. \end{array}$$

The projector *P* induces a splitting of the total Hilbert space H in two subspaces *P*H and (I−P)H, with the interaction term acting non-trivially only in the subspace *P*H. In the complementary subspace (I−P)H, the evolution is free for Fermionic particles. This means that solutions of the free theory are also solutions of the interacting one, as opposed to the Bosonic case for which the interaction is non-trivial also in (I−P)H.

## 5. Review of the Solutions

We focus in this section on the antisymmetric solutions of the Thirring walk that actually feel the interaction. From the remarks that we have made in the previous section, such solutions can only be found in the subspace *P*H. Formally, we have to solve the eigenvalue equation $PU_{2}\left(\chi ,p\right)|\psi \rangle =e^{-i\omega }|\psi \rangle$, with |ψ〉∈PH. Conveniently, we write a vector |ψ〉∈PH in the form

(25) $$|\psi \rangle =\sum_{z\in Z}\left(\begin{array}{c} \psi^{1}\left(z\right)\\ 0\\ 0\\ \psi^{4}\left(z\right) \end{array}\right)⊗|2z+1\rangle +\sum_{z\in Z}\left(\begin{array}{c} 0\\ \psi^{2}\left(z\right)\\ \psi^{3}\left(z\right)\\ 0 \end{array}\right)⊗|2z\rangle ,$$

and the antisymmetry condition becomes:

(26) $$\begin{array}{c} \psi^{1,4}\left(-z\right)=-\psi^{1,4}\left(z-1\right), \end{array}$$

(27) $$\begin{array}{c} \psi^{2}\left(-z\right)=-\psi^{3}\left(z\right). \end{array}$$

The restriction of the walk to the subspace *P*H entails that the eigenvalue problem is equivalent to the following system of equations:

(28) $$\left\{\begin{array}{c} e^{-i\omega }\psi^{1}\left(z\right)=\nu^{2}e^{i2p}\psi^{1}\left(z\right)-i\mu \nu e^{ip}e^{i\chi \delta_{z,0}}\psi^{2}\left(z\right)-i\mu \nu e^{ip}e^{i\chi \delta_{z,-1}}\psi^{3}\left(z+1\right)-\mu^{2}\psi^{4}\left(z\right),\\ e^{-i\omega }\psi^{2}\left(z\right)=-i\mu \nu e^{ip}\psi^{1}\left(z-1\right)+\nu^{2}e^{i\chi \delta_{z,1}}\psi^{2}\left(z-1\right)-\mu^{2}e^{i\chi \delta_{z,0}}\psi^{3}\left(z\right)-i\mu \nu e^{-ip}\psi^{4}\left(z-1\right),\\ e^{-i\omega }\psi^{3}\left(z\right)=-i\mu \nu e^{ip}\psi^{1}\left(z\right)-\mu^{2}e^{i\chi \delta_{z,0}}\psi^{2}\left(z\right)+\nu^{2}e^{i\chi \delta_{z,-1}}\psi^{3}\left(z+1\right)-i\mu \nu e^{-ip}\psi^{4}\left(z\right),\\ e^{-i\omega }\psi^{4}\left(z\right)=-\mu^{2}\psi^{1}\left(z\right)-i\mu \nu e^{-ip}e^{i\chi \delta_{z,0}}\psi^{2}\left(z\right)-i\mu \nu e^{-ip}e^{i\chi \delta_{z,-1}}\psi^{3}\left(z+1\right)+\nu^{2}e^{-i2p}\psi^{4}\left(z\right). \end{array}\right.$$

The most general solution of Equation (28) for p∉{0,π/2} has two forms:

(29) $$\begin{array}{c} U_{2}\left(\chi ,p\right)|\psi_{\pm \infty }\rangle =e^{\pm i2p}|\psi_{\pm \infty }\rangle ,\;\psi_{\pm \infty }\left(z\right)=\left\{\begin{array}{cc} \left(\begin{array}{c} \zeta_{\pm \infty }\\ \eta_{\pm \infty }\\ -\eta_{\pm \infty }\\ \zeta_{\pm \infty }^{\prime } \end{array}\right)\delta_{z,0}, & z\geq 0,\\ \mathrm{antisymmetrized}, & z<0, \end{array}\right. \end{array}$$

and

(30) $$\begin{array}{c} \begin{array}{c} \psi \left(z\right)=\left\{\begin{array}{cc} \sum_{s,r=\pm }\int_{S}\;dk\;g_{\omega }^{sr}\left(k\right)w_{k}^{sr}\left(z\right), & z>0,\\ \mathrm{antisymmetrized}, & z<0, \end{array}\right.\;\;w_{k}^{sr}\left(z\right):=\left(\begin{array}{c} v_{k}^{sr,1}e^{-i(2z+1)k}\\ v_{k}^{sr,2}e^{-i(2z)k}\\ v_{k}^{sr,3}e^{-i(2z)k}\\ v_{k}^{sr,4}e^{-i(2z+1)k} \end{array}\right),\\ \psi \left(0\right)=\left(\begin{array}{c} \sum_{s,r=\pm }\int_{S}\;dk\;g_{\omega }^{sr}\left(k\right)v_{k}^{sr,1}\\ \xi \\ -\xi \\ \sum_{s,r=\pm }\int_{S}\;dk\;g_{\omega }^{sr}\left(k\right)v_{k}^{sr,4} \end{array}\right), \end{array} \end{array}$$

with $k=k_{R}+ik_{I}$, $S:=\left\{\;k\in C|k_{R}\in \left(-\pi ,\pi \right]\;\right\}$, and $g_{\omega }^{sr}$ satisfying the condition

(31) $$\begin{array}{c} e^{-i\omega }\neq e^{-i\omega_{sr}\left(p,k\right)}\Rightarrow g_{\omega }^{sr}\left(k\right)=0. \end{array}$$

Solving Equation (28) corresponds now to find the function $g_{\omega }^{sr}$. Let us now study the equation

(32) $$e^{-i\omega_{sr}\left(p,k\right)}=e^{-i\omega }.$$

Since $e^{-i\omega_{sr}\left(p,k\right)}$ has to be an eigenvalue of $U_{2}\left(\chi ,p\right)$, $\omega_{sr}\left(p,k\right)$ must be real and thus $k\in \Gamma_{f}$ or $k\in \Gamma_{l}$ with l=0,±1,2, so we conveniently define the sets:

(33) $$\begin{array}{c} \Omega_{f}^{sr}\left\{\;e^{-i\omega_{sr}\left(p,k\right)}\;|\;k\in \Gamma_{f}\;\right\},\;\Omega_{l}^{sr}\left\{\;e^{-i\omega_{sr}\left(p,k\right)}\;|\;k\in \Gamma_{l}\;\right\}, \end{array}$$

(34) $$\begin{array}{c} \Gamma_{f}\left\{\;k\in S\;|\;k_{I}=0\;\right\},\;\Gamma_{l}\left\{\;k\in S\;|\;k_{R}=l\frac{\pi }{2}\;\right\},\;l=0,\pm 1,2. \end{array}$$

It is easy to see that $\Omega_{f}^{sr}\cap \Omega_{l}^{sr}=\emptyset$ for all *s*, *r* and *l*, and the range of the function $e^{-i\omega_{sr}\left(p,k\right)}$ covers the entire unit circle except for the points $e^{\pm i2p}$. Therefore, we can discuss separately the case $e^{-i\omega }\in \Omega_{f}^{sr}$ and the case $e^{-i\omega }\in \Omega_{l}^{sr}$. A solution with $e^{-i\omega }=e^{\pm i2p}$ actually exists, corresponding to the function of Equation (29), and it will be discussed in Section 5.3.

Let us start with the case $e^{-i\omega }\in \Omega_{f}^{sr}$, which will lead to the characterization of the continuous spectrum of the Thirring walk $U_{2}\left(\chi ,p\right)$ and of the scattering solutions.

### 5.1. Scattering Solutions

**Figure 1:**
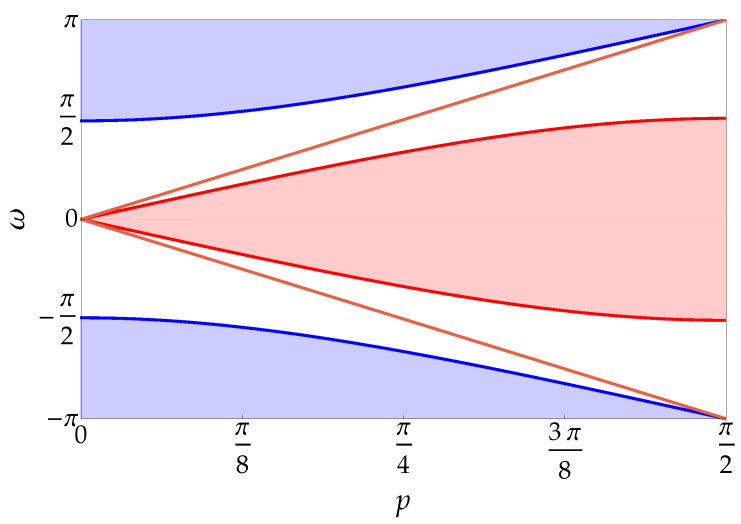
Continuous spectrum of the two-particle walk as a function of the total momentum p∈[0,π/2] with mass parameter m=0.7. The continuous spectrum is the same as in the free case. The solid blue curves are described by the functions ω=±2ω(p), and the red ones by ω=±(π−2Arccos(nsinp)). As one can notice, the light-red lines ω=±2p lie entirely in the gaps between the solid curves, highlighting the fact that $e^{\pm i2p}$ is not in the range of $e^{-i\omega_{sr}\left(p,k\right)}$ for p≠0,π/2 (see text).

**Figure 2:**
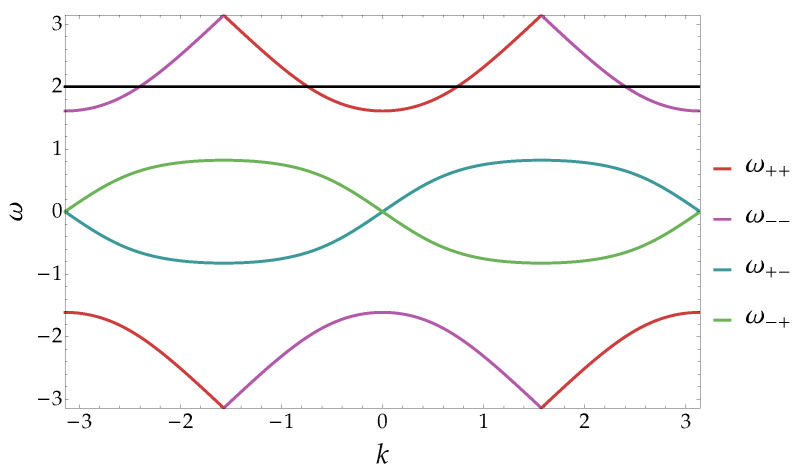
Spectrum of the walk for m=0.6 and p=π/6 as a function of *k*. The colours highlight the different ranges of eigenvalues corresponding to the dispersion relation $\omega_{sr}\left(p,k\right)$. The range of $\omega_{sr}\left(p,k\right)$ is understood to be computed mod(2π). One can notice that there are four values of the relative momentum *k* having the same value of the dispersion relation (ω=2 in the figure). This is in contrast to the Hamiltonian model for which there are only two solutions.

In this section, we assume p∉{0,π/2} with $e^{-i\omega }\in \Omega_{f}^{sr}$. This implies that $e^{-i\omega }\neq e^{\pm i2p}$: indeed, as one can notice from Figure 1, the lines ω=±2p lie entirely in the gaps between the curves ω=±2ω(p) and ω=±(π−2Arccos(nsinp)). The solution is thus the one given in Equation (30). One can prove that $\Omega_{f}^{++}=\Omega_{f}^{--}$ and $\Omega_{f}^{+-}=\Omega_{f}^{-+}$. Furthermore, as one can notice from Figure 2, there are four values of the triple (s,r,k) such that $e^{-i\omega_{sr}\left(p,k\right)}=e^{-i\omega }$ for a given value of $e^{-i\omega }$: if the triple (+,+,k) is a solution, so are (+,+,π−k), (−,−,−k) and (−,−,k−π); and if (+,−,k) is a solution, then also (+,−,π−k), (−,+,−k) and (−,+,k−π) are solutions. This result greatly simplifies Equation (30). Indeed, the sum over s,r and the integral over *k* reduces to the sum of four terms:

(35) $$\begin{array}{cc} \psi_{k}^{\pm ,1}\left(z\right):=\left(\alpha_{k}^{\pm }v_{k}^{+\pm ,1}+\delta_{k}^{\pm }v_{k-\pi }^{-\mp ,1}\right)e^{-i(2z+1)k}-\left(\beta_{k}^{\pm }v_{-k}^{\pm +,1}+\gamma_{k}^{\pm }v_{\pi -k}^{\mp -,1}\right)e^{i(2z+1)k},\; & z\geq 0,\\ \psi_{k}^{\pm ,2}\left(z\right):=\left(\alpha_{k}^{\pm }v_{k}^{+\pm ,2}-\delta_{k}^{\pm }v_{k-\pi }^{-\mp ,2}\right)e^{-i2zk}-\left(\beta_{k}^{\pm }v_{-k}^{\pm +,2}-\gamma_{k}^{\pm }v_{\pi -k}^{\mp -,2}\right)e^{i2zk},\; & z>0,\\ \psi_{k}^{\pm ,3}\left(z\right):=\left(\alpha_{k}^{\pm }v_{k}^{+\pm ,3}-\delta_{k}^{\pm }v_{k-\pi }^{-\mp ,3}\right)e^{-i2zk}-\left(\beta_{k}^{\pm }v_{-k}^{\pm +,3}-\gamma_{k}^{\pm }v_{\pi -k}^{\mp -,3}\right)e^{i2zk},\; & z>0,\\ \psi_{k}^{\pm ,4}\left(z\right):=\left(\alpha_{k}^{\pm }v_{k}^{+\pm ,4}+\delta_{k}^{\pm }v_{k-\pi }^{-\mp ,4}\right)e^{-i(2z+1)k}-\left(\beta_{k}^{\pm }v_{-k}^{\pm +,4}+\gamma_{k}^{\pm }v_{\pi -k}^{\mp -,4}\right)e^{i(2z+1)k}, & z\geq 0,\\ \psi_{k}^{\pm ,2}\left(0\right)=-\psi_{k}^{\pm ,3}\left(0\right)\xi . \end{array}$$

As we will see, the original problem can be simplified in this way to an algebraic problem with a finite set of equations. We note that the fact that the equation $e^{-i\omega_{sr}\left(p,k\right)}=e^{-i\omega }$ has a finite number of solutions is a consequence of the fact that we are considering a model in one spatial dimension. However, in analogous one-dimensional Hamiltonian models (e.g., the Hubbard model), the degeneracy of the eigenvalues is two.

Let us consider for the sake of simplicity the solution of the kind $\psi_{k}^{+,j}\left(z\right)$, since the other one can be analysed in a similar way. Using the notation of Appendix, Equation (35) reduces to the expressions (dropping the + superscript)

(36) $$\begin{array}{c} \psi_{k}^{1}\left(z\right)=a\left[\lambda e^{-i(2z+1)k}-\rho e^{i(2z+1)k}\right],\\ \psi_{k}^{2}\left(z\right)=\lambda be^{-i2zk}-\rho ce^{i2zk},\\ \psi_{k}^{3}\left(z\right)=\lambda ce^{-i2zk}-\rho be^{i2zk},\\ \psi_{k}^{4}\left(z\right)=d\left[\lambda e^{-i(2z+1)k}-\rho e^{i(2z+1)k}\right],\\ \lambda :=\alpha_{k}+\delta_{k},\;\rho :=\beta_{k}+\gamma_{k},\\ \psi_{k}^{2}\left(0\right)=\xi . \end{array}$$

We notice that now the number of unknown parameters is further reduced to three, namely λ, ρ, and ξ. Clearly, one of the parameters can be fixed by choosing arbitrarily the normalization. From now on, we fix λ=1 and define $T_{+}:=\rho$. Equation (36) has to satisfy the recurrence relations of Equation (28) for z=0 and z=1, while, for z>1, it is automatically satisfied. For z=0, Equation (28) becomes

(37) $$\begin{array}{c} e^{-i\omega }\psi_{k}^{1}\left(0\right)=\nu^{2}e^{i2p}\psi_{k}^{1}\left(0\right)-i\mu \nu e^{ip}e^{i\chi }\xi -i\mu \nu e^{ip}\psi_{k}^{3}\left(1\right)-\mu^{2}\psi_{k}^{4}\left(0\right), \end{array}$$

(38) $$\begin{array}{c} e^{-i\omega }\xi =i\mu \nu e^{ip}\psi_{k}^{1}\left(0\right)-\nu^{2}\psi_{k}^{3}\left(1\right)-\mu^{2}e^{i\chi }\xi +i\mu \nu e^{-ip}\psi_{k}^{4}\left(0\right), \end{array}$$

(39) $$\begin{array}{c} -e^{-i\omega }\xi =-i\mu \nu e^{ip}\psi_{k}^{1}\left(0\right)-\mu^{2}e^{i\chi }\xi +\nu^{2}\psi_{k}^{3}\left(1\right)-i\mu \nu e^{-ip}\psi_{k}^{4}\left(0\right), \end{array}$$

(40) $$\begin{array}{c} e^{-i\omega }\psi_{k}^{4}\left(0\right)=-\mu^{2}\psi_{k}^{1}\left(0\right)-i\mu \nu e^{-ip}e^{i\chi }\xi -i\mu \nu e^{-ip}\psi_{k}^{3}\left(1\right)+\nu^{2}e^{-i2p}\psi_{k}^{4}\left(0\right). \end{array}$$

Starting from Equation (37), we can notice that $\nu^{2}e^{i2p}a-i\mu \nu e^{ip}e^{ik}b-i\mu \nu e^{ip}e^{-ik}c-\mu^{2}d=e^{-i\omega }a$, where we employed the notation of Appendix A, so that we obtain $\xi =e^{-i\chi }\left(b-T_{+}c\right)$. We can then substitute this expression in Equation (39) and use the relations

(41) $$\begin{array}{c} -i\mu \nu e^{ip}e^{ik}a+\nu^{2}e^{i2k}b-\mu^{2}c-i\mu \nu e^{-ip}e^{ik}d=e^{-i\omega }b, \end{array}$$

(42) $$\begin{array}{c} -i\mu \nu e^{ip}e^{-ik}a-\mu^{2}b+\nu^{2}e^{-i2k}c-i\mu \nu e^{-ip}e^{-ik}d=e^{-i\omega }c, \end{array}$$

to obtain the expression

(43) $$\begin{array}{c} 1-1e^{-i\chi }\left(b-T_{+}c\right)=T_{+}b-c, \end{array}$$

and thus

(44) $$T_{+}=\frac{c+e^{-i\chi }b}{b+e^{-i\chi }c}=\frac{g_{+}\left(p+k\right)+e^{-i\chi }g_{+}\left(p-k\right)}{g_{+}\left(p-k\right)+e^{-i\chi }g_{+}\left(p+k\right)}.$$

For these values of ξ and $T_{+}$ one can verify that Equation (28) is satisfied also for z=1, thus concluding the derivation. For the solution of the kind $\psi_{k}^{-,j}\left(z\right),$ we can follow a similar reasoning, obtaining the analogous quantity $T_{-}$:

(45) $$T_{-}:=\frac{g_{+}\left(p+k\right)+e^{-i\chi }g_{-}\left(p-k\right)}{g_{-}\left(p-k\right)+e^{-i\chi }g_{+}\left(p+k\right)}.$$

It is worth noticing that $T_{\pm }$ is of unit modulus for k∈(−π,π].

The final form of the solution results in being:

(46) $$\begin{array}{c} \psi_{k}^{\pm ,1}\left(z\right)=\left(v_{k}^{+\pm ,1}+v_{k-\pi }^{-\mp ,1}\right)e^{-i(2z+1)k}-T_{\pm }\left(v_{-k}^{\pm +,1}+v_{\pi -k}^{\mp -,1}\right)e^{i(2z+1)k},\\ \psi_{k}^{\pm ,2}\left(z\right)=e^{-i\chi \delta_{z,0}}\left[\left(v_{k}^{+\pm ,2}-v_{k-\pi }^{-\mp ,2}\right)e^{-i2zk}-T_{\pm }\left(v_{-k}^{\pm +,2}-v_{\pi -k}^{\mp -,2}\right)e^{i2zk}\right],\\ \psi_{k}^{\pm ,3}\left(z\right)=\left(v_{k}^{+\pm ,3}-v_{k-\pi }^{-\mp ,3}\right)e^{-i2zk}-T_{\pm }\left(v_{-k}^{\pm +,3}-v_{\pi -k}^{\mp -,3}\right)e^{i2zk},\\ \psi_{k}^{\pm ,4}\left(z\right)=\left(v_{k}^{+\pm ,4}+v_{k-\pi }^{-\mp ,4}\right)e^{-i(2z+1)k}-T_{\pm }\left(v_{-k}^{\pm +,4}+v_{\pi -k}^{\mp -,4}\right)e^{i(2z+1)k}, \end{array}$$

which in terms of the relative coordinate *y* can be written as

(47) $$\psi_{k}^{\pm }\left(y\right)=\left\{\begin{array}{cc} e^{-i\chi \delta_{z,0}\delta_{j,2}}\left[\left(v_{k}^{+\pm }+v_{k-\pi }^{-\mp }\right)e^{-iky}-T_{\pm }\left(v_{-k}^{\pm +}+v_{\pi -k}^{\mp -}\right)e^{iky}\right], & y\geq 0,\\ \mathrm{antisymmetrized}, & y<0. \end{array}\right.$$

We can interpret such a solution as a scattering of plane waves for which the coefficient $T_{\pm }$ plays the role of the transmission coefficient. Being the total momentum a conserved quantity, the two particles can only exchange their momenta, as expected from a theory in one dimension. Furthermore, for each value *k* of the relative momentum, the two particles can also acquire an additional phase of π. As the interaction is a compact perturbation of the free evolution, the continuous spectrum is the same as that of the free walk. Equation (46) provides the generalized eigenvector if $U_{2}\left(\chi ,p\right)$ corresponding to the continuous spectrum $\sigma_{c}=\Omega_{f}^{++}\cup \Omega_{f}^{+-}$.

### 5.2. Bound States

In the previous section, we derived the solutions in the continuous spectrum, which can be interpreted as scattering plane waves in one spatial dimension. We seek now the solutions corresponding to the discrete spectrum, namely solutions with eigenvalues in any one of the sets $\Omega_{l}^{sr}$. The derivation of the solution follows similar steps as for the scattering solutions. In particular, the degeneracy in *k* is the same: there are four solutions to the equation $e^{-i\omega_{sr}\left(p,k\right)}=e^{-i\omega }$ even in this case, as proved in [45]. Therefore, the general form of the solution in this case can be written again as in Equation (35) and, following the same reasoning, one obtains the same set of solutions as in Equation (46). At this stage, we did not impose that the solution is a proper eigenvector in the Hilbert space H. To this end, we have to set $T_{\pm }=0$ to eliminate the exponentially-divergent terms in Equation (46). As one can prove, the equation $T_{\pm }=0$ has only one solution for fixed values of χ and *p*. More precisely, there is a unique $k\in \Gamma_{0}\cup \Gamma_{-1}\cup \Gamma_{1}\cup \Gamma_{2}$, with $k_{I}<0$ and $e^{i\chi }\notin \left\{1,-1\right\}$, such that either $T_{+}=0$ or $T_{-}=0$.

In other words, for each pair of values (χ,p), the walk $U_{2}\left(p\right)$ has one and only one eigenvector corresponding to an eigenvalue in the point spectrum. Such eigenvector can be written as

(48) $$\begin{array}{c} \psi_{\tilde{k}}^{1}\left(z\right)=\left(v_{\tilde{k}}^{+\pm ,1}+v_{\tilde{k}-\pi }^{-\mp ,1}\right)e^{-i\left(2z+1\right)\tilde{k}},\\ \psi_{\tilde{k}}^{2}\left(z\right)=e^{-i\chi \delta_{z,0}}\left[\left(v_{\tilde{k}}^{+\pm ,2}-v_{\tilde{k}-\pi }^{-\mp ,2}\right)e^{-i2z\tilde{k}}\right],\\ \psi_{\tilde{k}}^{3}\left(z\right)=\left(v_{\tilde{k}}^{+\pm ,3}-v_{\tilde{k}-\pi }^{-\mp ,3}\right)e^{-i2z\tilde{k}},\\ \psi_{\tilde{k}}^{4}\left(z\right)=\left(v_{\tilde{k}}^{+\pm ,4}+v_{\tilde{k}-\pi }^{-\mp ,4}\right)e^{-i\left(2z+1\right)\tilde{k}}, \end{array}$$

where $\tilde{k}$ is the solution of $T_{+}=0$ or $T_{-}=0$ and ± chosen accordingly. More compactly, in the *y* coordinate, the solution can be written as

(49) $$\psi_{\tilde{k}}\left(y\right)=\left\{\begin{array}{cc} e^{-i\chi \delta_{z,0}\delta_{j,2}}\left[\left(v_{\tilde{k}}^{+\pm }+v_{\tilde{k}-\pi }^{-\mp }\right)e^{-i\tilde{k}y}\right], & y\geq 0,\\ \mathrm{antisymmetrized}, & y<0. \end{array}\right.$$

**Figure 3:**
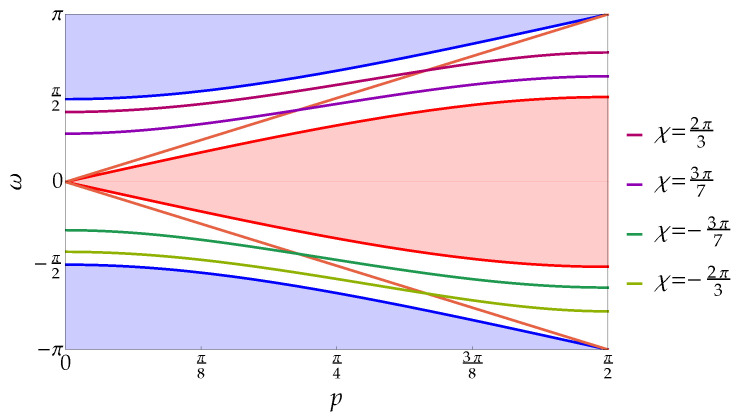
Complete spectrum of the two-particle Thirring walk as a function of the total momentum *p* with mass parameter m=0.7. The continuous spectrum is as in Figure 1. The solid lines in the gaps show the point spectrum for different values of the coupling constant: from top to bottom, χ=2π/3,3π/7,−3π/7,−2π/3. It is worth noticing that, for each pair (χ,p), there is only one value in the discrete spectrum. The light-red lines ω=±2p lie entirely in the gap between the continuous bands highlighting the fact that the $e^{\pm i2p}$ is not in the range of $e^{-i\omega_{sr}\left(p,k\right)}$ for p≠0,π/2; for a given coupling constant χ, $e^{\pm i2p}$ is an eigenvalue for p=χ/2.

In Figure 3, the discrete spectrum of the interacting walk together with the continuous spectrum as a function of the total momentum *p* is depicted. The solid curves in the gaps between the continuous bands denote the discrete spectrum for different values of the coupling constant χ=2π/3,3π/7,−3π/7,−2π/3. Molecule states appear also in the Hadamard walk with the same on-site interaction [44].

**Figure 4:**
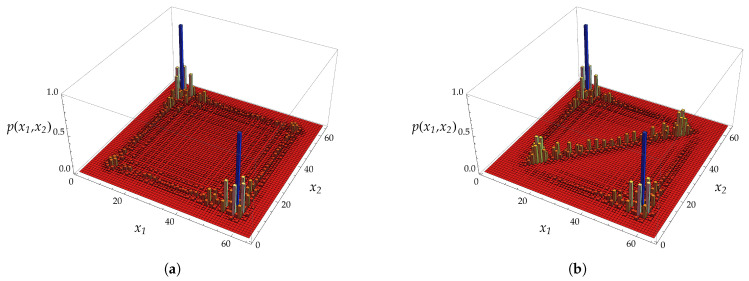
We show for comparison the free evolution (**a**) and the interacting one (**b**) highlighting the appearance of bound states components along the diagonal, namely when the two particles are at the same site (i.e., $x_{1}=x_{2}$), where $x_{1}$ and $x_{2}$ denote the positions of the two particles. The plots show the probability distribution $p(x_{1},x_{2})$ in position space after t=32 time-steps. The chosen value of the mass parameter is m=0.6 and the coupling constant is χ=π/2. The two particles are initially prepared in a singlet state located at the origin.

Referring to Figure 4, we show the evolution of two particles initially prepared in a singlet state localized at the origin. From the figure, one can appreciate the appearance of the bound state component that has non-vanishing overlapping with the initial state. The bound state, being exponentially decaying in the relative coordinate *y*, is localized on the diagonal of the plot, that is when the two particles lie at the same point.

**Figure 5:**
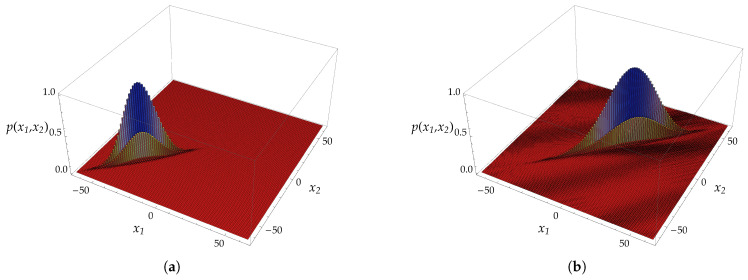
We show the evolution of a bound state of the two particles peaked around the value of the total momentum p=0.035π. The mass paramater is m=0.6 and the coupling constant χ=0.2π. In (**a**) is depicted the probability distribution of the initial state. In (**b**) is depicted the probability distribution of the evolved state after t=128 time-steps. One can notice that, in the relative coordinate $x_{1}-x_{2}$, the probability distribution remains concentrated on the diagonal, highlighting the fact that the two particles are in a bound state. The diffusion of the state happens only in the centre of a mass coordinate.

In Figure 5, the probability distribution of the bound state corresponding to a choice of parameters χ=0.2π and p=0.035π is depicted. The plot highlights the exponential decay of the tails, which is the characterizing feature of the bound state.

### 5.3. Solution for $e^{-i\omega }=e^{\pm i2p}$

Thus far, we have studied proper eigenvectors that decay exponentially as the two particles are further apart. However, the previous analysis failed to cover the particular case when $e^{-i\omega }=e^{\pm i2p}$, since the range of $e^{-i\omega_{sr}\left(p,k\right)}$ does not include the two points of the unit circle $e^{\pm i2p}$.

We now study the solutions with $e^{-i\omega }=e^{\pm i2p}$ having the form given in Equation (29). One can prove that such solutions are non-vanishing only for z=0 on *P*H, namely we look for a solution of the form

(50) $$\begin{array}{c} |\psi \rangle =\left(\begin{array}{c} -\zeta \\ 0\\ 0\\ -\zeta^{\prime } \end{array}\right)⊗|-1\rangle +\left(\begin{array}{c} 0\\ \eta \\ -\eta \\ 0 \end{array}\right)⊗|0\rangle +\left(\begin{array}{c} \zeta \\ 0\\ 0\\ \zeta^{\prime } \end{array}\right)⊗|1\rangle . \end{array}$$

Subtracting the first and the last equations of (28) using (50), we obtain the following equation:

(51) $$\left(e^{-i\omega }-e^{i2p}\right)\zeta =e^{i2p}\left(e^{-i\omega }-e^{-i2p}\right)\zeta^{\prime }.$$

If both ζ and $\zeta^{\prime }$ are non-zero, one can prove that a solution does not exist and thus we have to consider the two cases ζ=0 and $\zeta^{\prime }=0$ separately. Starting from $\zeta^{\prime }=0$, Equation (51) imposes that $e^{-i\omega }=e^{i2p}$, meaning that, if a solution exists in this case, it is an eigenvector corresponding to the eigenvalue $e^{i2p}$. From the second equation of (28), we obtain the relation

(52) $$\left(1-\mu^{2}e^{i(\chi -2p)}\right)\eta =i\mu \nu e^{-ip}\zeta$$

and, using the first equation of (28), it turns out that a solution exists only if $e^{i\chi }=e^{i2p}$, as expected, since, otherwise, the case of Section 5.2 would have held. The other case, namely $e^{-i\omega }=e^{-i2p}$, can be studied analogously. Let us, then, denote as $|\psi_{\pm \infty }\rangle$ such proper eigenvectors with eigenvalue $e^{\pm i2p}$ for $\chi =e^{\pm i2p}$ and, choosing $\eta =\frac{\mu }{\nu }$ as the value for the free parameter η, we obtain the following expression for $|\psi_{\pm \infty }\rangle$:

(53) $$\begin{array}{c} |\psi_{\pm \infty }\rangle =ie^{\pm ip}\left(\begin{array}{c} \frac{1\pm 1}{2}\\ 0\\ 0\\ -\frac{-1\pm 1}{2} \end{array}\right)⊗|-1\rangle +\left(\begin{array}{c} 0\\ \frac{\mu }{\nu }\\ -\frac{\mu }{\nu }\\ 0 \end{array}\right)⊗|0\rangle +ie^{\pm ip}\left(\begin{array}{c} -\frac{1\pm 1}{2}\\ 0\\ 0\\ \frac{-1\pm 1}{2} \end{array}\right)⊗|1\rangle . \end{array}$$

Such solutions provide a special case of molecule states (namely, proper eigenvectors of $U_{2}\left(\chi ,p\right)$), being localized on few sites, and differ from the previous solutions showing an exponential decay in the relative coordinate.

### 5.4. Solutions for p∈{0,π/2}

**Figure 6:**
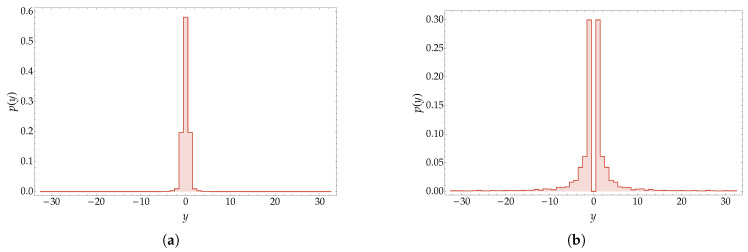
We show the case of two proper eigenstates for p=0. In both cases the mass parameter is *m* = 0.6. (**a**): probability distribution in the relative coordinate *y* of $\int \;dk\;\left(v_{k}^{+-}-v_{k}^{-+}\right)e^{-iyk}$. (**b**): probability distribution in the *y*-coordinate of $\int \;dk\;\left(v_{k}^{+-}+v_{k}^{-+}\right)e^{-iyk}$.

The solutions that we presented in the previous discussion do not cover the extreme values p=0,π/2 (see [45] for a reference). Let us consider for definiteness the case p=0, since the other case is obtained in a similar way. For $e^{-i\omega }\neq 1$, the previous analysis still holds. Indeed, noticing that $\omega_{\pm \pm }\left(0,k\right)=\pm 2\omega \left(k\right)$, we have ω(k)∈R and ω(k)≠0 if and only if $k\in \Gamma_{f}\cup \Gamma_{0}\cup \Gamma_{2}$, whereas $\omega_{\pm \mp }\left(0,k\right)=0$ for all k∈C. This means that the solutions $|\psi_{k}^{+}\rangle$ of Equation (46) are actually eigenvectors of $U_{2}\left(\chi ,0\right)$. Thus, the spectrum is made by a continuous part, given by the arc of the unit circle containing −1 and having $e^{\pm 2i\omega (0)}$ as extremes, and a point spectrum with two points: $e^{-2i\omega (\tilde{k})}$, where $\tilde{k}$ is the solution of $T_{+}=0$ for p=0, and 1. As shown in [45], 1 is a separated part of the spectrum of $U_{2}\left(\chi ,0\right)$ and the corresponding eigenspace is a separable Hilbert space of stationary bound states. This fact underlines an important feature of the Thirring walk not shared by analogous Hamiltonian models. It is remarkable that this behaviour occurs also for the free walk with χ=0. In Figure 6, we show the probability distribution of two states having the properties hereby discussed. It is worth noticing that all the states $v_{k}^{+-}$ with k∈(−π,π] are eigenvectors relative to the eigenvalue 1, and thus they generate a subspace on which the walk acts identically. We remark that this behaviour relies on the fact that the dispersion relation in one dimension is an even function of *k*.

## 6. Conclusions

In this work, we reviewed the Thirring quantum walk [45], providing a simplified derivation of its solutions for Fermionic particles. The simplified derivation relies on the symmetric properties of the walk evolution operator, allowing for separating the subspace of solutions affected by the interaction from the subspace where the interaction step acts trivially. The interaction term is the most general number-preserving interaction in one dimension, whereas the free evolution is provided by the Dirac QW [17].

We showed the explicit derivation of the scattering solutions (solutions for the continuous spectrum) as well as for the bound-state solutions. The Thirring walk features also localized bound states (namely, states whose support is finite on the lattice) when $e^{-i\omega }=e^{\pm i2p}$. Such solutions exist only when the coupling constant is χ=2p. Figure 4 depicts the evolution of a perfectly localized state showing the overlapping with bound state components. In Figure 5, we reported the evolution of a bound state of the two particles peaking around a certain value of the total momentum: one can appreciate that the probability distribution remains localized on the main diagonal during the evolution.

Finally, we showed that bound states exist also for a vanishing coupling constant—even though this is true only for a finite set of values of the total momentum *p*—which is a striking difference between the discrete model of the present work and corresponding Hamiltonian systems.

## Appendix A. Notation

For the single particle walk of Equation (3), the eigenstates can be written as

(A1) $$v_{p}^{s}=\frac{1}{|N_{s}\left(p\right)|}\left(\begin{array}{c} -i\mu \\ g_{s}\left(p\right) \end{array}\right),\;g_{s}\left(p\right):=-i\left(s\sin\omega \left(p\right)+\nu \sin p\right),$$

with $|N_{s}{\left(p\right)|}^{2}=\mu^{2}+{\left|g_{s}\left(p\right)\right|}^{2}$. For the two-particle walk, we define $v_{k}^{rs}:=v_{p+k}^{s}⊗v_{p-k}^{r}$. If s=r, then we name the related eigenspace the *even* eigenspace; whereas, if s≠r, we call the related eigenspace the *odd* eigenspace. As proven in item 3 of Lemma 1 of [45], for a given *k*, the degeneracy is 4 both in the even and in the odd case. Namely, if the triple (+,+,k) is a solution, then also (+,+,−k), (−,−,π−k) and (−,−,k−π) are solutions; if the triple (+,−,k) is a solution, then also (+,−,π−k) and (−,+,k−π) are solutions.

Explicitly, for the even case, we have:

(A2) $$\begin{array}{cc} v_{k}^{++}\propto \left(\begin{array}{c} -\mu^{2}\\ -i\mu g_{+}\left(p-k\right)\\ -i\mu g_{+}\left(p+k\right)\\ g_{+}\left(p+k\right)g_{+}\left(p-k\right) \end{array}\right), & v_{\pi -k}^{--}\propto \left(\begin{array}{c} -\mu^{2}\\ i\mu g_{+}\left(p+k\right)\\ i\mu g_{+}\left(p-k\right)\\ g_{+}\left(p+k\right)g_{+}\left(p-k\right) \end{array}\right), \end{array}$$

(A3) $$\begin{array}{cc} v_{-k}^{++}\propto \left(\begin{array}{c} -\mu^{2}\\ -i\mu g_{+}\left(p-k\right)\\ -i\mu g_{+}\left(p+k\right)\\ g_{+}\left(p+k\right)g_{+}\left(p-k\right) \end{array}\right), & v_{k-\pi }^{--}\propto \left(\begin{array}{c} -\mu^{2}\\ i\mu g_{+}\left(p-k\right)\\ i\mu g_{+}\left(p+k\right)\\ g_{+}\left(p+k\right)g_{+}\left(p-k\right) \end{array}\right). \end{array}$$

Analogously for the odd case, the eigenstates are

(A4) $$\begin{array}{cc} v_{k}^{+-}\propto \left(\begin{array}{c} -\mu^{2}\\ -i\mu g_{-}\left(p-k\right)\\ -i\mu g_{+}\left(p+k\right)\\ g_{+}\left(p+k\right)g_{-}\left(p-k\right) \end{array}\right), & v_{\pi -k}^{+-}\propto \left(\begin{array}{c} -\mu^{2}\\ i\mu g_{+}\left(p+k\right)\\ i\mu g_{-}\left(p-k\right)\\ g_{+}\left(p+k\right)g_{-}\left(p-k\right) \end{array}\right), \end{array}$$

(A5) $$\begin{array}{cc} v_{-k}^{-+}\propto \left(\begin{array}{c} -\mu^{2}\\ -i\mu g_{+}\left(p+k\right)\\ -i\mu g_{-}\left(p-k\right)\\ g_{-}\left(p+k\right)g_{+}\left(p-k\right) \end{array}\right), & v_{k-\pi }^{-+}\propto \left(\begin{array}{c} -\mu^{2}\\ i\mu g_{+}\left(p+k\right)\\ i\mu g_{-}\left(p-k\right)\\ g_{+}\left(p+k\right)g_{-}\left(p-k\right) \end{array}\right). \end{array}$$

In order to simplify the derivation of the solution, we adopt the following notation:

(A6) $$\begin{array}{cccc} v_{k}^{++}=:\left(\begin{array}{c} a\\ b\\ c\\ d \end{array}\right), & v_{-k}^{++}=\left(\begin{array}{c} a\\ c\\ b\\ d \end{array}\right), & v_{\pi -k}^{--}=\left(\begin{array}{c} a\\ -c\\ -b\\ d \end{array}\right), & v_{k-\pi }^{--}=\left(\begin{array}{c} a\\ -b\\ -c\\ d \end{array}\right), \end{array}$$

(A7) $$\begin{array}{cccc} v_{k}^{+-}=:\left(\begin{array}{c} a^{\prime }\\ b^{\prime }\\ c^{\prime }\\ d^{\prime } \end{array}\right), & v_{-k}^{-+}=\left(\begin{array}{c} a^{\prime }\\ c^{\prime }\\ b^{\prime }\\ d^{\prime } \end{array}\right), & v_{\pi -k}^{+-}=\left(\begin{array}{c} a^{\prime }\\ -c^{\prime }\\ -b^{\prime }\\ d^{\prime } \end{array}\right), & v_{k-\pi }^{-+}=\left(\begin{array}{c} a^{\prime }\\ -b^{\prime }\\ -c^{\prime }\\ d^{\prime } \end{array}\right). \end{array}$$
